# *Notes from the Field:* Reemergence of *Mycoplasma pneumoniae* Infections in Children and Adolescents After the COVID-19 Pandemic, United States, 2018–2024

**DOI:** 10.15585/mmwr.mm7307a3

**Published:** 2024-02-22

**Authors:** Chris Edens, Benjamin R. Clopper, Jourdan DeVies, Alvaro Benitez, Erin R. McKeever, Dylan Johns, Bernard Wolff, Rangaraj Selvarangan, Jennifer E. Schuster, Geoffrey A. Weinberg, Peter G. Szilagyi, Fatimah S. Dawood, Lakshmi Radhakrishnan, Christina Quigley, Leila C. Sahni, Natasha Halasa, Laura S. Stewart, Meredith L. McMorrow, Brett Whitaker, Danielle M. Zerr, Vasanthi Avadhanula, John V. Williams, Marian G. Michaels, Aaron Kite-Powell, Janet A. Englund, Mary Allen Staat, Kathleen Hartnett, Heidi L. Moline, Adam L. Cohen, Maureen Diaz

**Affiliations:** ^1^Division of Bacterial Diseases, National Center for Immunization and Respiratory Diseases, CDC; ^2^Coronavirus and Other Respiratory Viruses Division, National Center for Immunization and Respiratory Diseases, CDC; ^3^Detect & Monitor Division, Office of Public Health Data, Surveillance, and Technology, CDC; ^4^Maximus Federal Services, Inc., McLean, Virginia; ^5^ICF, Atlanta, Georgia; ^6^Children's Mercy Hospital, Kansas City, Missouri; ^7^Department of Pediatrics, University of Rochester School of Medicine and Dentistry, Rochester, New York; ^8^Cincinnati Children’s Hospital Medical Center, Cincinnati, Ohio; ^9^Texas Children's Hospital and Baylor College of Medicine, Houston, TX; ^10^Vanderbilt University Medical Center, Nashville, Tennessee; ^11^Seattle Children’s Research Institute, Seattle Washington; ^12^University of Pittsburgh Medical Center, Children's Hospital of Pittsburgh, Pittsburgh Pennsylvania.

**Summary**

**What is already known about this topic?**

*Mycoplasma pneumoniae* is a common cause of mild respiratory illness, though severe infection can lead to pneumonia. Resistance to macrolides, the recommended treatment, is widespread in Asia though uncommon in the United States. *M. pneumoniae* infections decreased globally during the COVID-19 pandemic.

**What is added by this report?**

Data from the National Syndromic Surveillance Program and the New Vaccine Surveillance Network showed an increase in *M. pneumoniae* in the United States beginning in fall 2023, though below prepandemic levels.

**What are the implications for public health practice?**

Providers might consider *M. pneumoniae* during fall and winter respiratory illness seasons. Macrolides remain the first-line treatment for *M. pneumoniae* infections in the United States.

*Mycoplasma pneumoniae* is a common cause of respiratory infections, particularly in school-aged children. Most infections display as a mild respiratory illness sometimes referred to as “walking pneumonia.” However, some persons experience severe pneumonia and require hospitalization. Significant cyclical increases in *M. pneumoniae* infections have been observed every 3–5 years, likely because of changes in the predominant circulating strain (*1*). *M. pneumoniae* infections are typically treated using macrolide antibiotics. Macrolide resistance varies globally, with the highest resistance prevalence (>90%) in Asia (*2*). After implementation of nonpharmaceutical interventions in response to COVID-19, the frequency of identified *M. pneumoniae* infections substantially declined beginning in 2020 (*3*). This pattern was also observed for other respiratory pathogens. Beginning in the fall of 2023, China and other countries identified a reemergence of this bacterium (*2*,*4*).

Using data from CDC’s National Syndromic Surveillance Program (NSSP),* the percentage of *M. pneumoniae*–related diagnoses among all pneumonia emergency department visits were compared before, during, and after the COVID-19 pandemic. Data from the New Vaccine Surveillance Network (NVSN)^†^ were analyzed to compare the percentage of positive *M. pneumoniae* laboratory test results in the United States during the same periods. During September 2023–January 2024, 14 *M. pneumoniae*–positive specimens collected at four NVSN sites were sent to CDC for molecular testing to identify common genetic changes that confer macrolide resistance. This activity was reviewed by CDC, deemed not research, and was conducted consistent with applicable federal law and CDC policy.^§^

## Investigation and Outcomes

NSSP includes *International Classification of Diseases, Tenth Revision* (ICD-10) diagnostic codes from more than 6,500 emergency departments (ED) and urgent care facilities located in all 50 states, the District of Columbia, and Guam. For this analysis, data from NSSP were restricted to ED visits by children and adolescents. NVSN conducts prospective, active, population-based surveillance among children and adolescents for acute respiratory illness^¶^ at seven U.S. pediatric medical centers. All children enrolled in NVSN from four sites received *M. pneumoniae*–inclusive multi-pathogen panel testing, and enrollees from the other three sites had *M. pneumoniae* test results included if conducted for diagnostic purposes. Three periods were defined and analyzed: January 2018–April 2020 (prepandemic period), May 2020–August 2023 (pandemic period), and September 2023–December 2023 (postpandemic period).

NSSP data** were searched for ED visits with a diagnosis of pneumonia^††^ with *M. pneumoniae*–related diagnostic codes (ICD-10 J15.7 and J20.0^§§^). The percentage of *M. pneumoniae*–related diagnoses among pneumonia ED visits reported in NSSP decreased from 1.15% (4,681 of 407,514) during the prepandemic period to 0.35% (1,233 of 355,508) during the pandemic period and then increased to 0.89% (597 of 66,736) during the postpandemic period. Similarly, the percentage of test results within the NVSN network that were positive for *M. pneumoniae* decreased from 1.2% (165 of 13,800) during the prepandemic period to 0.04% (10 of 24,256) during the pandemic period and then increased to 0.53% (13 of 2,470) during the postpandemic period (Figure). Fourteen *M. pneumoniae*–positive specimens collected at four NVSN sites during September 2023–January 2024 were sent to CDC for macrolide resistance testing. Among 14 specimens, 13 were determined to be susceptible to macrolides. 

**FIGURE:**
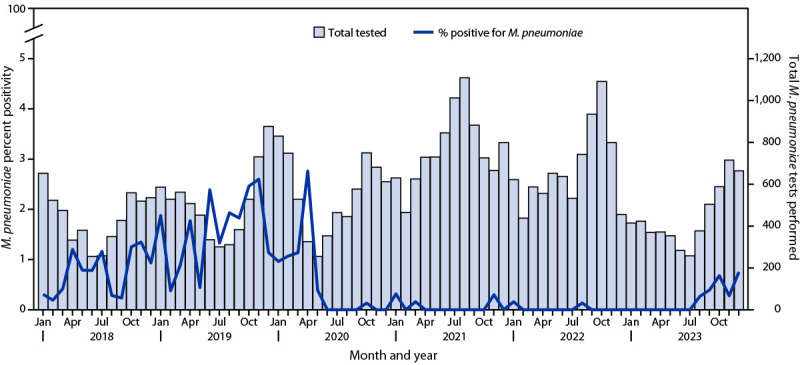
Monthly number of *Mycoplasma pneumoniae *tests performed and percentage of positive test results among children and adolescents with acute respiratory illness — four sites, New Vaccine Surveillance Network, 2018–2023

## Preliminary Conclusions and Actions

Data collected by NSSP and NVSN demonstrate that the percentage of *M. pneumoniae* diagnoses and positive *M. pneumoniae* test results decreased during the COVID-19 pandemic. The percentage of diagnoses and positive test results have increased since September 2023 but remain below prepandemic levels. Among the small number of specimens available for testing, resistance to macrolides was uncommon. This report highlights the need for continued surveillance for *M. pneumoniae* infections and macrolide-resistant *M. pneumoniae* in the United States. Providers should consider *M. pneumoniae* as part of the differential diagnosis for cases of community-acquired pneumonia during fall and winter respiratory illness seasons. Despite ongoing concerns regarding antimicrobial resistance, macrolides remain the recommended first-line treatment for *M. pneumoniae* infections in the United States (*5*).

## Footnotes

* https://www.cdc.gov/nssp/overview.html

† https://www.cdc.gov/surveillance/nvsn/index.html

§ 45 C.F.R. part 46.102(l)(2), 21 C.F.R. part 56; 42 U.S.C. Sect. 241(d); 5 U.S.C. Sect. 552a; 44 U.S.C. Sect. 3501 et seq.

¶ Acute respiratory illness is defined as the occurrence of one or more of the following symptoms during a defined period before the enrollment encounter (<14 days during January 2018–October 2022 or ≤10 days during November 2022–December 2023): fever, cough, earache, nasal congestion, runny nose, sore throat, vomiting after coughing, wheezing, shortness of breath, rapid or shallow breathing, apnea, apparent life-threatening event, or brief resolved unexplained event.

** NSSP emergency department data come from a substantial percentage of emergency departments in the United States. However, the number of facilities sharing data has changed over time. To reduce the potential impact of changes in reporting patterns, analyses were restricted to facilities consistently reporting an average weekly informative discharge diagnosis of ≥75% and with a coefficient of variation ≤40. Among all emergency departments sending data to CDC, 46% consistently reported more complete data and had at least one pneumonia emergency department visit during 2018–2023.

†† Influenza due to identified novel influenza A virus with pneumonia (J09.X1); influenza due to other identified influenza virus with pneumonia (J10.00–01, J10.08); influenza due to unidentified influenza virus with pneumonia (J11.00, J11.08); viral pneumonia, not elsewhere classified (J12.0–.3, J12.8–.9); pneumonia due to *Streptococcus pneumoniae* (J13); pneumonia due to *Haemophilus influenzae* (J14); bacterial pneumonia, not elsewhere classified (J15.0–9); pneumonia due to other infectious organisms, not elsewhere classified (J16.0, J16.8); pneumonia in diseases classified elsewhere (J78); J18.0 bronchopneumonia, unspecified organism (J18.0–2; J18.8–.9); acute respiratory distress syndrome (J80); Legionnaires disease (A48.1); and acute bronchitis due to *Mycoplasma pneumoniae* (J20.0).

§§ J.15.7: Pneumonia due to *Mycoplasma pneumoniae*; J20.0: acute bronchitis due to *Mycoplasma pneumoniae*.
